# What Does the Population Attributable Fraction Mean?

**Published:** 2006-12-15

**Authors:** Beverly Levine

**Affiliations:** Department of Public Health Education, University of North Carolina at Greensboro, Greensboro, NC

## Abstract

Recent controversy over the disagreement of population attributable fraction estimates for the obesity–total mortality relation has made the concept of attributable fraction visible in both scientific and popular news. Most of the attention in writings on the attributable fraction has focused on technical matters of estimation and on ensuring a causal relationship between exposure and outcome. Yet some of the most illuminating questions about the attributable fraction have to do with another causal question and how the measure is to be interpreted in light of the answer to this question: *What interventions are available to cause the assumed reduction in risk among the exposed and the consequent estimated reduction in disease burden?* In this paper, I discuss the limitations to the common interpretations of the attributable fraction and argue that these limitations cannot be overcome merely by better statistical modeling or by use of better data sets. They must be addressed through discussion of specific interventions and the hypothesized causal consequences of such specified interventions.

## Introduction

Recent controversy over the accuracy of population attributable fraction (AF) estimates for the obesity–total mortality relation has made the concept of AF (also called *attributable risk*) highly visible in both scientific and popular news. Both the Institute of Medicine (1) and the Centers for Disease Control and Prevention (CDC) (2) have sponsored recent workshops on the topic of how best to estimate the effects of obesity on the risk of mortality in the United States. and how to resolve disagreements over published estimates (3,4). Many scientific resources have been directed toward this topic, and the discussion has been published in top medical and scientific journals in the United States (3-6).

This article will not address the political or scientific aspects of this controversy. Its purpose is to discuss the general use of the AF estimate as a practical tool in applied epidemiology and public health.

## Definition

The AF is formally written as

P(D) - P(D | Ē)P(D)

where *P*(*D*) is the (unconditional) probability of disease over a specified time period, and *P*(*D *| *Ē*) is the probability of disease over the same time period conditional on nonexposed status (not exposed to the risk factor under study). The AF is the difference between overall average risk of the entire population (both exposed and unexposed people) and average risk in the unexposed, expressed as a fraction of the overall average risk.

Depending on the types of data available, there are different formulas used to estimate the AF. Much of the discussion in epidemiology textbooks, in the section on AF in the *Encyclopedia of Biostatistics* (7,8), and in articles on AF in epidemiologic and biostatistical journals is devoted to the technical topic of choosing the most appropriate formula for estimating the above fraction, given various constraints, once it can be assumed that there is a causal relationship between exposure and disease. Yet some of the most interesting questions about the AF have to do with another causal question that cannot be answered through recourse to technical discussion: *What interventions are available to cause the assumed reduction in risk among the exposed and the consequent estimated reduction in disease burden?* Such a question is rarely, if ever, discussed in writings on the AF.

Before addressing the central point — that this other causal question is critical to the significance of the AF — I first discuss the two most common interpretations of the AF. These interpretations, although related, are not equivalent. First, the AF is widely interpreted as the proportion of disease burden causally explained by, or attributable to, the risk factor(s) being considered. Second, the AF is the proportion of disease risk that would be eliminated from the population if exposure to the risk factor were eliminated.

### The AF as a partitioning of causality

The interpretation of the AF as the proportion of disease burden attributable to a factor (or a set of factors) is commonly used by those who wish to differentiate between the portion of disease risk that is understood and the portion that remains to be understood. This interpretation has been used in breast cancer. For example, reports of AFs of about 25% for the major breast cancer risk factors have been used to imply that 75% of the disease of breast cancer is not understood or is not attributable to known causes (8). This interpretation is also sometimes used by genetic epidemiologists to estimate what proportion of disease is causally attributable to genes (9-11). With AFs such as these, no interventions are intended. The fractions are estimated for the purpose of summarizing and partitioning causal knowledge — often between known and unknown causes, as has been the case in breast cancer — or between genetic and nongenetic causes.

Underlying this interpretation is the philosophical question of what we mean when we say that a certain percentage of disease in the population is *caused by*, *attributable to*, or *explainable by* a given risk factor or set of factors.

Greenland and Robins (12) tackle the issue of what is meant by the phrase *attributable to* (5) when they draw a distinction between *excess* and *etiologic* cases. They provide a thorough discussion of the difference between these kinds of cases and show why the AF will usually greatly underestimate the proportion of disease burden that is etiologically related to the exposure.

Another concern with the interpretation of the AF as the proportion of disease caused by an exposure stems from the model of causes that underlies much of epidemiology. This model of sufficient component causes holds that a given case of disease could theoretically have been averted over a considered time period if *any one* of a sufficient set of causes were averted. The AF for different exposures considered one at a time will usually sum to greater than 100% (greater than the total number of cases) for a given outcome. In the single-factor-at-a-time AF analytic method, a death or a case of disease (e.g., myocardial infarction) attributable to exposure X (e.g., hypertension) could also be, and often is, attributable to exposure Y (e.g., elevated cholesterol levels). Thus, the consideration of an outcome as attributable to (or caused by) exposure X (rather than Y) is often arbitrary.

A third reason to question the use of the AF in causal partitioning is that a large AF may reflect merely a broad exposure definition rather than any valuable understanding about causality. As an extreme example of this, consider that one could report an AF of 100% if one were to consider age >15 years as a risk factor for breast cancer. This would say nothing about causality. As Wacholder et al (13) demonstrate, the AF will always increase with a broader definition of exposure provided that the individuals newly included under the broader definition have a relative risk for disease greater than 1.0 when compared with the remaining unexposed group. As an exposure definition is made more sensitive (i.e., broader), the AF will increase, but the absolute risk of disease in the exposed category will decline as long as there is a monotonic dose–response relationship between exposure level and risk of disease. For many scientists, it is a high absolute risk of disease rather than a broad exposure definition (and high AF) that is key to valuable information about causality.

Interpretation of the AF as a partition delineating what proportion of disease or mortality risk scientists should consider causally related and causally unrelated to a given factor is problematic. Kempthorne, in a classic *Biometrics* paper (14), argued against any attempt to quantitatively partition causality when multiple factors or forces determine the outcome. He stated that the results of such partitioning attempts are meaningless for understanding causal processes and for considering realistic effects of intervention.

### The AF as proportion of preventable disease

The AF is frequently interpreted as the proportion of disease risk or incidence that could be eliminated from the population if exposure were eliminated. The expectation is that the AF has a practical value for those interested in public health prevention policy, particularly when dealing with an exposure that is modifiable.

When the AF is interpreted as the proportion of disease risk that could be eliminated from the population if exposure were eliminated, the simple fraction is interpreted as an answer to the following narrow, precise question:

*What proportion of disease risk could be eliminated if absolute risk in the exposed were to suddenly and sustainably go to the level of absolute risk in the unexposed, while nothing else, including absolute risk in the unexposed, were to change?*

This question subsumes another more common, narrower question:

*What proportion of disease risk could be eliminated if exposure were to be eliminated, while nothing else changed?*

Given the algebraic structure of the AF, the modifiability (or elimination) of exposure is not the key criterion. The key is elimination of excess risk associated with exposure, which can theoretically happen in various ways besides actual elimination of exposure.

A rephrasing of the questions in the previous example is helpful because it points out the severe limitation to the interpretation of the AF as a proportion of disease risk that can be eliminated. The question,

*What proportion of disease risk could be eliminated if the absolute risk in the exposed were to suddenly and sustainably go to the level of absolute risk in the unexposed, while nothing else, including absolute risk in the unexposed, were to change?*

is an interesting and valuable question only if one can also ask and answer the following question:

*What intervention is available to cause the disease risk in the exposed to quickly become that of the unexposed, while simultaneously changing nothing else?*

If this second question sounds meaningless in a given situation — perhaps because no such intervention nor anything close has been proved — I would argue that the interpretation of the AF as the proportion of disease risk that can be eliminated is also meaningless because the fundamental assumption underlying the AF, that disease risk in the exposed immediately becomes that of the unexposed, is impossible to meet.

It is an irony that in all the discussions about AF, the causality question that has received the most attention is whether or not there is truly a causal relationship between exposure and outcome. An example is the discussion about AF in the *Encyclopedia of Biostatistics* (7) in which the three conditions that must be met for the AF to be interpreted as the proportion of disease risk that can be eliminated are the following: 1) the estimation of the AF is unbiased; 2) the exposure is causal rather than merely associated with disease; and 3) elimination of the risk factor has to have no effect on the distribution of other risk factors. If one cannot assume a causal relationship between exposure and disease, calculation of the AF has no clear value. It is also true, however, that there is an equally important question of causality that needs to be addressed if the above interpretation of the AF is to have any meaning: *What intervention is available to cause the assumed reduction in disease risk?* This question has received scant, if any, attention in the literature on attributable fraction. Yet we have data available in many situations where an AF is estimated to at least begin to address this question.

Returning to the specific topic that began this article — AF estimation for the obesity and mortality association — suppose there were a scientific consensus that the prevalence of obesity could be greatly reduced in the United States. Different interventions to achieve this reduction would have different effects on the burden of mortality. Hernan (15) points out that the notion of *causal effect* is not well defined unless one can specify an intervention, even a hypothetical one, to eliminate the cause. He notes that the value of the counterfactual outcome (which in the obesity–mortality AF situation is the number of deaths that would be eliminated following the elimination of obesity) depends entirely on the actual intervention used to manipulate exposure. A strategy to eliminate (or greatly reduce) the prevalence of obesity in the United States. that relied upon successful persuasion of overweight and obese individuals in the population to adopt eating and activity patterns that led to safe and sustainable weight loss would have very different consequences for public health and mortality than a strategy that relied on widespread use of gene therapy or liposuction to eliminate excess weight. These planned interventions would have different consequences from a catastrophic event that resulted in a great reduction in prevalence of overweight and obesity. None of these hypothetical interventions necessarily has its causal effect captured in the obesity–mortality AF estimate.

Some have used the AF to rank order exposures in terms of their hypothetical public health priority even if there is no available or proposed intervention. For example, if the AF estimate for risk factor X is higher than that for risk factor Y, a conclusion might be that risk factor X is the more burdensome exposure and should receive more attention from a prevention standpoint. But issues of available or potential interventions, the risks and benefits of such interventions, and the relation of the exposure to other exposures in the population (i.e., is it feasible to hypothesize about changing the exposure while holding all other risk factors unchanged?) must be rigorously addressed before one can assume that an exposure with a higher AF is more important for policy makers to consider than another exposure. The topic of how public health priorities should be set is beyond the scope of this article, but Buchanan presents a thought-provoking discussion relevant to this complex topic (16).

## Conclusion

As discussed previously in this article and as stated by Kempthorne (14), attempts to partition causality when multiple forces act together to produce the outcome are meaningless. With respect to interpretation of an AF as the proportion of disease risk that could be eliminated if the excess risk associated with exposure were to be eliminated, there may be valuable meaning under a specific set of assumptions. In addition to the assumptions commonly listed in textbooks, there is one more critical assumption: that we can envision a specific intervention that will cause the estimated reduction in risk in the exposed while changing no other risk factor distributions.

Some might argue that in the absence of this last assumption, the AF nonetheless allows for an interesting theoretical case study (i.e., what would happen to the disease burden if we *were* to find and use such an intervention?). Because such theoretical cases are not subject to tests of falsifiability, we must ask ourselves rigorously, in each case, what purpose they serve. For many exposures, it is time for more complex and specific theoretical case studies than simple AF estimation. These more complex theoretical experiments would hypothesize about effects of specific interventions to reduce or eliminate exposure risk in specific populations and subpopulations by using the diverse data gained from public health activities. In the work of Berry et al (17), there is elegant precedent for such complex thought experiments and for the careful use of existing data to draw as precise a conclusion as possible about the public health consequences of specific interventions.

The AF is only a simple fraction derived from the arithmetic manipulation of probabilities. As with many other measures in public health, how this fraction is interpreted is key. In some settings it has taken on a life of its own, regardless of its meaning in reality. The burden is on those providing AF estimates to state what their value is to public health professionals and policy makers. The rest of us in the public health community have the responsibility to continually draw the discussion of AF estimates back to the central question of public health implications.

This paper is not an argument for never computing a population AF. It is an argument for more clarity, justification, and complex thinking when using this measure. AFs are only a beginning of the discussion of the public health consequences of intervening to reduce the prevalence of risk exposures.
